# Brazilian Academy of Cardiovascular Surgery: From Idea to Reality

**DOI:** 10.21470/1678-9741-1-2020-0631

**Published:** 2020

**Authors:** José Wanderley Neto, Enio Buffolo, José Teles de Mendonça

**Affiliations:** 1 Ex-Presidente da Sociedade Brasileira de Cirurgia Cardiovascular Brasileira (SBCCV), São Paulo, SP, Brazil.; 2 Diretor do Instituto de Doenças do Coração de Alagoas (IDC), Maceió, AL, Brazil.; 3 Prof. Titular da Discipline of Cardiovascular Surgery, Hospital São Paulo, Escola Paulista de Medicina, Universidade Federal de São Paulo - EPM-UNIFESP, São Paulo, SP, Brazil.; 4 Cardiovascular Surgery Division, Universidade Federal de Sergipe (UFS), Aracaju, SE, Brazil.

## “Knowledge is the paradox in which dividing is multiplying.”

During the Heart Transplantation Symposium in Maceió, in October 2019, the idea of creating the Brazilian Academy of Cardiovascular Surgery was born and aimed at documenting, for future generations of cardiac surgeons, the rich history of cardiac surgery in Brazil.

First-generation Brazilian cardiac surgeons were not just spectators^[1]^, but participants in the development of surgical procedures in the treatment of coronary artery disease^[2,3]^, heart failure^[4,5]^, valvular heart surgery^[6,7]^, congenital heart surgery^[8-10]^ and many others^[11,12]^. In addition, they developed expertise in the manufacture of medical equipment in Brazil, such as heart lung machine^[13,14]^, oxygenators^[15,16]^, pacemakers^[17]^, valves^[18-20]^, stents^[21]^ and many others^[1]^. This allowed to overcome the difficult trade barrier for materials and equipment necessary for the development of the specialty at that time, easing costs of procedures and enabling the application of techniques and concepts from developed countries. Surgical equipment and supplies manufactured in Brazil were soon recognized for their excellent quality. They were subsequently exported and used in many countries in Latin America and other continents, contributing to the development of cardiac surgery in these countries and helping to save lives^[22]^.

When one reads articles such as “Cardiovascular Surgery in Brazil: Achievements and possibilities”, written by Euryclides de J. Zerbini, about a symposium held in 1963, with the participation os Dr. Zerbini and Antonio C. de Azevedo, Cid Nogueira, Domingo J. de Moraes, Hugo J. Felipozzi, Delmond Bittencourt, Adib D. Jatene and Jesse Teixeira^[1]^, “History of World Cardiac Surgery” by Domingo Braile and Moacyr Godoy^[23]^, “Short History of Cardiac Surgery: and everything happened before our eyes” by Paulo Prates^[24]^ and “History of Brazilian Cardiac Surgery” by Iseu Affonso da Costa^[25]^, one can witness the development of what is now considered one of the most beautiful, recent and complex medical specialties: cardiovascular surgery.

The courage and determination of cardiologists and cardiac surgeons in Brazil were the same as in the more developed countries. This resulted in the development and application of technology and procedures with minimal delay in Brazil^[26]^. Great enthusiasm and momentum at the time further enhanced their creativity, contributing greatly to technical innovations in the world of cardiac surgery^[19]^.

These masters also emphasized that medicine is the most wonderful and enriching of all professions, and that it is complete when practiced with passion. It must also be practiced with continuous and inexhaustible affection, solidarity, empathy and compassion^[27]^.

Traditionally, an academy differs from association, society or syndicate. The academy is aimed at preservation of history, science, legacy for young surgeons, and does not represent any government or opposition. The academy emphasizes knowledge and human beings, participating in the teaching of medicine at different stages of a surgeons’ career. It also constitutes, in a particular way, the universality of science^[28]^.

One of the pre-requisites to become a member of the Brazilian Academy of Cardiovascular Surgery is to have at least 30 years of clinical practice. This allows for contribution to the development and teaching in a specialty that has had glorious but also challenging and difficult moments. This knowledge and experience will then be transmitted to in-training cardiac surgeons, through meetings, in which invitees can present historical perspectives from professors. There will also be sessions aimed at recognizing those who contributed to the development of our specialty. Occasionally, there will be opportunities to hold sessions aimed at the general population, presented by special guests.

The basic foundation of the Brazilian Academy of Cardiovascular Surgery is original, because it is aimed at cultural differentiation of a specialty, and does not have the format of the National Academy of Medicine, or State Academies, from Brazil, which brings together all other medical specialties.

The rescue of history is also fundamental for medicine, as stated by Emile Paul Littre: “if medical science is not to be lowered to office level, consideration should be given to its history and old monuments from the past”^[29]^.

The Academy will not compete or occupy the space naturally dedicated to the Brazilian Society of Cardiovascular Surgery and will have no political intentions. The objective of the Academy is to complement medical knowledge through cultural exchange. This will be the goal of the Brazilian Academy of Cardiovascular Surgery, honoring our dear friend, Domingo Marcolino Braile, who represented the ethos of the Brazilian Academy, and should have been its first president.

“The supreme science is history, because it is the only work built by men.” (Giambattista Vico, Principi de una Scienza Nuova, 1725)^[29]^.

Signed by 5 former presidents of the SBCCV*, 3 full members**, 1 past president in memoriam***, and 3 international honorary members****

Domingo Marcolino Braile***

Enio Buffolo*

Fabio Biscegli Jatene*

Fernando Antonio Lucchese**

José Wanderley Neto*

José Teles de Mendonça*

Manuel de Jesus Antunes****

Mozart Augusto Soares de Escobar**

Paulo Roberto Slud Brofman*

Ricardo de Carvalho Lima**

Rodolfo A. Neirotti****

Tomas Antonio Salerno****

## Figures and Tables

**Figure:**
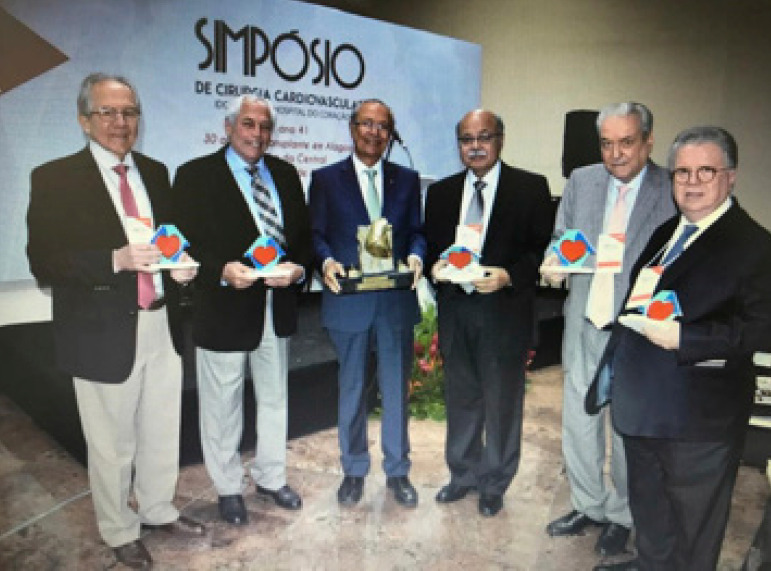
Ricardo Lima, Mozart Escobar, José Wanderley Neto, José Teles, Ênio Buffolo and Fernando Lucchese, Foundation of the Brazilian Academy of Cardiovascular Surgery, Maceió, Brazil, 2019.
